# Influence of Pickling Process on *Allium cepa* and *Citrus limon* Metabolome as Determined *via* Mass Spectrometry-Based Metabolomics

**DOI:** 10.3390/molecules24050928

**Published:** 2019-03-07

**Authors:** Mohamed A. Farag, Ahmed F. Tawfike, Marwa S. Donia, Anja Ehrlich, Ludger A. Wessjohann

**Affiliations:** 1 Pharmacognosy department, College of Pharmacy, Cairo University, Kasr el Aini St., Cairo 11562, Egypt; 2 Department of Chemistry, School of Sciences & Engineering, The American University in Cairo, New Cairo 11835, Egypt; 3 Department of Computational and Analytical Science, Rothamsted Research, Harpenden AL5 2JQ, UK; ahmed.tawfike@rothamsted.ac.uk; 4 Department of Pharmacognosy, Faculty of Pharmacy, Helwan University, Cairo 11795, Egypt; 5 Pharmacognosy Department, College of Pharmacy, Suez Canal University, Ismailia 41522, Egypt; mdonia00@gmail.com; 6 Leibniz Institute of Plant Biochemistry, Department of Bioorganic Chemistry, Weinberg 3, D-06120 Halle (Saale), Germany; Anja.Ehrlich@ipb-halle.de

**Keywords:** *Allium cepa* red cv., *Citrus limon*, pickling, volatiles, SPME, chemometrics

## Abstract

Brine, the historically known food additive salt solution, has been widely used as a pickling media to preserve flavor or enhance food aroma, appearance, or other qualities. The influence of pickling, using brine, on the aroma compounds and the primary and secondary metabolite profile in onion bulb *Allium cepa* red cv. and lemon fruit *Citrus limon* was evaluated using multiplex metabolomics technologies. In lemon, pickling negatively affected its key odor compound “citral”, whereas monoterpene hydrocarbons limonene and γ-terpinene increased in the pickled product. Meanwhile, in onion sulphur rearrangement products appeared upon storage, i.e., 3,5-diethyl-1,2,4-trithiolane. Profiling of the polar secondary metabolites in lemon fruit via ultra-performance liquid chromatography coupled to MS annotated 37 metabolites including 18 flavonoids, nine coumarins, five limonoids, and two organic acids. With regard to pickling impact, notable and clear separation among specimens was observed with an orthogonal projections to least squares-discriminant analysis (OPLS-DA) score plot for the lemon fruit model showing an enrichment of limonoids and organic acids and that for fresh onion bulb showing an abundance of flavonols and saponins. In general, the pickling process appeared to negatively impact the abundance of secondary metabolites in both onion and lemon, suggesting a decrease in their food health benefits.

## 1. Introduction

Pickling—using food additives, i.e., a highly concentrated salt solution (brine)—is a well-known method for preserving foods including vegetables that has been used for thousands of years and remains in use today. Undoubtedly, the most unambiguous role of pickling for human nutrition is to make the nutrients naturally present in the original food materials more palatable while also preserving their quality. Nevertheless, pickling processes can have several effects on food nutritional qualities [1]. The effect of the pickling preservation method and storage time on vitamins and amino acid composition in garlic was reported by Montano et al., 2004 [2]. In addition, the influence of pickling with and without fermentation was revealed, with fermented products showing higher α-tocopherol, riboflavin, and total amino acid levels except for thiamine and arginine. Another study on Chinese cabbage reported that the nitrogenous content, viz. nitrate and nitrite, in vegetables was dramatically affected by fermentation [3]. Other studies [4,5] demonstrated a decrease in the level of nitrates during the natural fermentation of cabbage and radish. The risk of the reduction of nitrates to nitrite lies in the possible induction of methemoglobinemia [6]. The metabolic changes associated with pickling may thus develop some distinctive properties related to its organoleptic characters, shelf life, and/or safety [7,8]. In terms of human health benefits, pickled foods produced by fermentation have been recognized as protective for the gut [9] via the microbial and enzymatic processing of food ingredients. Fermentation can improve protein and fiber digestibility by improving micronutrient bioavailability and decaying anti-nutritional factors [10]. Fermentation processes can also reduce toxic compounds such as aflatoxins concurrent with the production of antimicrobial factors such lactic acid, bacteriocins, carbon dioxide, and ethanol, which prevent the growth of food-borne pathogens [11,12].

*Citrus* fruits are enriched in polyphenols which mainly include flavanones and, to a lesser extent, flavones, flavonols, coumarins, and phenolic acids, viz. ferulic, *p*-coumaric, sinapic, and caffeic acids [13]. Phenolic compounds are well known for their myriad health benefits, as they exhibit antioxidant, anticancer, and anti-inflammatory properties, mostly attributed to their remarkable free radical scavenging activities [14]. A study on four Tunisian *Citrus* varieties revealed the abundance of phenolic acids (73.13–86.40%) [15] among their secondary metabolite profiles, whereas camphene was found to be a major component in *C. limon*. *Citrus* fruit is also abundant in coumarins, limonoids, sterols, and flavonoids, which all exhibit various bioactivities [16].

Onion bulbs (*Allium cepa*) encompass a myriad of bioactive natural product classes which are linked to its health benefits, i.e., anticancer or chemo-preventive agents [17]; antimicrobial [18], antiviral [19], and antioxidant activities [20]; protective activities against cardiovascular diseases [21], diabetes [22], neurodegenerative disorders, and cataract formation [23,24]. Among the secondary metabolites found in onion, flavonoids, fructans, and organosulphur compounds were mainly found to mediate its health benefits [25,26,27]. We have previously reported on the flavor makeup of *A. cepa* red cv. grown in the Siwa Oasis, Egypt, revealing its enrichment in sulphur compounds [28]. Solid-phase microextraction (SPME) is regarded as a powerful analytical tool for volatiles extraction without solvent consumption and with no heat application, which aids in maintaining the original food aroma without the formation of artifacts as compared to steam distillation [29]. Further, confined direct MS analysis with real-time ion source and its applications in the analysis of volatile organic compounds of *Citrus limon* (lemon) and *Allium cepa* (onion) was reported [30]. In terms the secondary non-volatile metabolite composition of *A. cepa*, flavonols and acylated amino acids were the major forms as analyzed via UPLC-QTOFMS [28]. Metabolomics provides a holistic overview for the chemical profile of an organism (set of metabolites) exposed to different conditions. It can provide a shorter and more accessible route for visualizing metabolic difference in food matrices [31]. Significant strides have been made in the analysis of plant foods, mostly using different metabolomics platforms data [32]. The application of mass spectrometry (MS) coupled to an array of separation techniques [33,34] helps to simultaneously analyze a relatively large number of metabolites in a high-throughput, reproducible, and sensitive manner [35]. This study aims to explore the impact of pickling as a preservation method on the metabolite profile. Onion and lemon were employed as two different models considering their different compositional profiles, as these represent major food crops preserved using pickling. The effect of pickling, with and without fermentation, in terms of processing and storage time on organosulphur compounds of garlic has been previously reported [36]. This study reported the drawbacks of fermentation on the bioactive sulphur compounds, namely γ-glutamyl peptides and *S*-alk(en)yl-l-cysteine sulfoxides. For the comprehensive analysis of metabolites, solid-phase microextraction (SPME) coupled with GC-MS was employed for volatiles profiling, whereas UPLC-QTOFMS was used to capture the polar non-volatile secondary metabolites. Results from the current study reveal for the apparent negative effect of pickling on the bioactive and flavor chemical composition in both lemon and onion.

## 2. Results and Discussion

### 2.1. Profiling of Onion and Lemon Volatiles Using SPME/GC-MS and Multivariate Data Analysis

**Figure 1:**
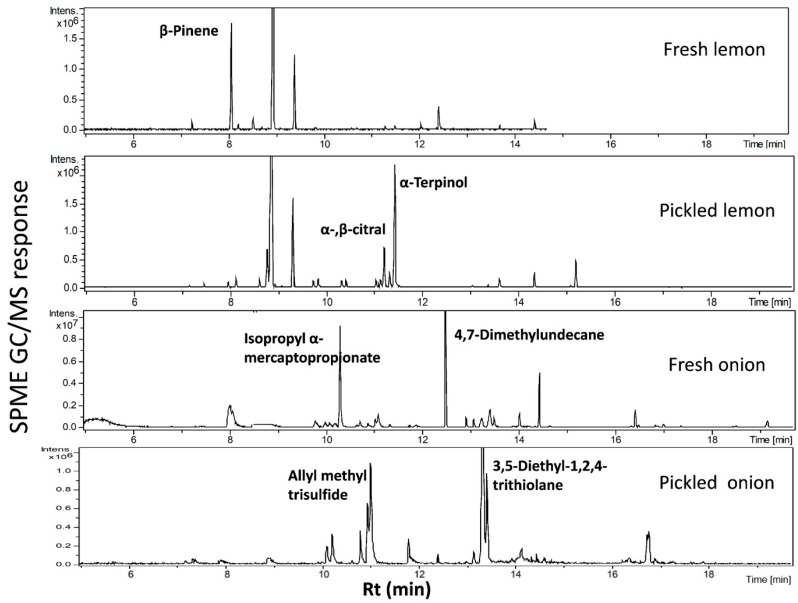
Solid-phase microextraction (SPME)/GC-MS chromatogram of headspace volatiles collected from fresh and pickled onion bulb and lemon fruit.

Three independent biological replicates representing each specimen including fresh and pickled samples were extracted and analyzed under identical conditions. The volatile analysis of lemon samples (Figure 1) revealed for the presence of 31 volatile components, with monoterpene hydrocarbons representing the major class at ca. 96.6% and 97.5% in the fresh and pickled lemon, respectively (Table 1). The fresh lemon monoterepenes pool was characterized by a relatively high β-pinene level (33%) versus an enrichment of limonene and δ-terpinene in pickled lemon. With regard to oxygenated monoterpenes, α/β-citral (odor key marker) was remarkably reduced in pickled lemon and likely accounted for the lower aroma strength of pickled lemon. Furthermore, total sesquiterpene hydrocarbons were found to be slightly higher in fresh lemon with (*E*)-α-bergamotene as major component compared with a relative increase of (*E*/*Z*)-α-farnesene and β-bisabolene in pickled lemon. SPME/GC-MS volatiles analysis of fresh and pickled *A. cepa* onion bulbs detected the presence of 20 volatile components belonging to both sulphur and non-sulphur compounds (Figure 1). Sulphur volatiles were dominant with 96.6% and 98.1% in pickled and fresh samples, respectively. Several of the volatile components either disappeared or were reduced in pickled onion, as shown in Table 2. Moreover, significant increases in the levels of allyl methyl trisulfide, 3,5-diethyl-1,2,4-trithiolane, and 2-hexyl-5-methyl-3(2*H*)-furanone were detected in pickled onion. Such chemical change in volatiles makeup due to pickling is likely to affect its organoleptic properties such as taste and aroma.

**Figure 2:**
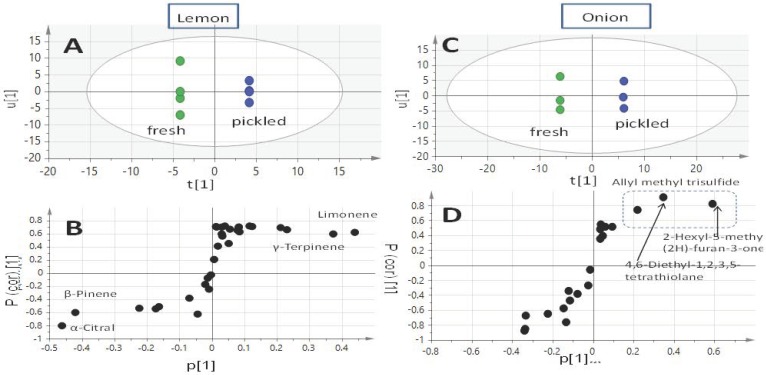
OPLS-DA score plot derived from modeling volatile metabolites. The metabolites were analyzed via SPME/GC-MS, and were derived fresh and pickled lemon fruit (**A**) and onion bulb (**C**), each modeled one at a time (*n* = 3). The respective *S*-plots, (**B**) and (**D**), show the covariance p[1] against the correlation p(cor)[1] of the variables of the discriminating component of the OPLS-DA model. Cut-off values of *p* < 0.05 were used; selected variables are highlighted in the *S*-plot and identifications are discussed in the text.

Multivariate data analysis was further applied to the volatiles abundance GC-MS dataset to visually demonstrate similarities and differences among specimens. Principal component analysis (PCA) was used as an unsupervised clustering model to highlight the variation based on volatiles abundance peak data. The PCA score plot (Figure S1) modeling fresh and pickled lemon showed no tight clustering of replicates, especially for pickled lemon. Consequently, supervised multivariate data analysis, i.e., OPLS, was employed for samples classification. OPLS-DA outscores PCA in the separation of the predictive variation from orthogonal variation and enhances data interpretation [37]. The OPLS-DA score plot (Figure 2A) showed better discrimination between the sample groups with the pickled group clustering separately from the fresh group. The S-loading plot derived from the OPLS-DA model is represented in Figure 2C. The figure highlights limonene and δ-terpinene as the most discriminatory aroma compounds for the pickled lemon, whereas β-pinene and α-citral were found distinctive for fresh lemon. Citral is a key flavor compound that accounts for lemon aroma [38]. Similarly, the OPLS-DA score plot of the pickled onion model (Figure 2C) discriminated between both sample groups, with 2-hexyl-5-methyl-3-(2*H*)-furanone and 3,5-diethyl-1,2,4-trithiolane (identified as markers for pickled onion) being highly correlated to the variation of the pickled onion. 2-Hexyl-5-methyl-3(2*H*)-furanone has previously been reported in “shallot” onion [39] as a decomposition fatty acid product derived from the onion lachrymatory factor [40], suggesting that sulphur compounds also undergo such degradation during pickling. Similarly, 3,5-diethyl-1,2,4-trithiolane may occur due to the thermal decomposition of cysteine [41], as reported in heat-prepared *Allium* essential oil [39].

### 2.2. Profiling of Onion and Lemon Secondary Metabolites via UPLC-QTOFMS and Multivariate Data Analysis

To assess changes in secondary metabolite compositions of brine-preserved lemon and onion, non-targeted profiling was conducted using ultra-performance liquid chromatography (UPLC) coupled to a photodiode array and high resolution QTOF-MS operated in the negative- and positive-ionization modes (Figure S2). It should be noted that extracts were analyzed in both positive and negative-ion electrospray ionization (ESI) MS modes (Supplementary Figure S2) as changes in ESI polarity can often circumvent or significantly alter competitive ionization and suppression effects, revealing otherwise suppressed metabolite signals. We have previously reported on the UPLC-MS characterization of onion bulb, as published elsewhere [28], and we now present an annotation of metabolites for lemon fruit. Compared to the positive-ion ESI mode, the negative-ion MS spectra revealed better sensitivity and more observable peaks in the case of lemon. Nevertheless, it did not yield as much fragmentation information as in the positive-ion mode. Thus, the positive-ion mode provided more structural context and the negative-ion mode greater sensitivity. In total, 14 peaks from lemon fruit were annotated based on their negative-ionization mass spectral data versus 32 in the positive-ion mode, see Table 3. This is the first profile of *C. limon* fruit methanol extract from Egypt obtained via a UPLC-QTOFMS platform and it is presented herein as part of this study towards the composition of fresh *C. limon*. LC-MS and NMR were reported for the metabolite profiling of citrus oil derived from Italy [42]. A representative UPLC-MS base peak chromatogram (BPC) of *C. limon* is shown in (Figure S2A). The identities, retention times (rt), UV characteristics, and observed molecular and fragment ions for individual secondary metabolites are presented in Table 3. A total of 49 metabolites were detected, of which 36 were annotated. Metabolites belonged to several natural product classes including flavonoids, limonoids, coumarins, and phenolic/organic acids.

#### 2.2.1. Phenolic Acids

As precursors for most phenolic metabolites, phenolic acids are commonly reported in metabolite profiling studies of functional foods, often conjugated with sugars as in the case of *C. limon*. Phenolic acids are also known to contribute, among others, to the flavor/taste of foods. In this study, two phenolic acid glycosides were found to be predominant in lemon fruit extracts, namely 6-*O*-Feruloyl-hexoside (peak 6, *m*/*z* 341.087 [M − H]^−^ and citrusin E (peak 24, *m*/*z* 353.0872 [M − H]^−^) preferentially ionized in negative-ionization mode.

#### 2.2.2. Flavonoids

Flavonoids were detected as the most abundant class, represented by 18 peaks belonging to flavanone, flavone, and flavonols subclasses, principally as *O*-glycosides. In MS analysis, the nature of the sugars could be revealed by the elimination of the sugar residue, which would have a mass of 162 amu (hexose; glucose or galactose), 146 amu (rhamnose), or 132 amu (pentose; xylose or arabinose). The MS spectrum interpretation allowed for the annotation of hesperitin signals (*m*/*z* 301.27, C_16_H_13_O_6_^−^) in peaks 37 and 39, luteolin (*m*/*z* 285.21, C_15_H_9_O_6_^−^) in peaks 7 and 23, apigenin (*m*/*z* 269.21, C_15_H_9_O^−^) in peak 13, diosmetin (*m*/*z* 299.26, C_16_H_11_O_6_^−^) in peak 14, and limocitirin (*m*/*z* 345.21, C_17_H1_3_O_8_^−^) in peaks 17, 18, and 26. The readily cleaved sugar moieties from aglycone yielded a fragment mass in these respective peaks. In contrast, several flavonoid peaks (8–12) showed intense molecular ion peaks, with [M − 90 − H]^−^ and [M − 120 − H]^−^ fragments exclusively in flavone peaks of the MSn spectra indicative of sugar cleavage in *C*-glycoside peaks 8–12. The fragmentation pathways most characteristic of *C*-glycosyl flavonoids include dehydration (−18 amu) and cross-ring cleavage (0,2 and 0,3) of the sugar moiety, i.e., −120 amu and −90 amu for the *C*-hexosides; −90 amu and −60 amu for the *C*-pentosides. Citrus fruits are rich dietary sources of flavonoids [43] and thus exhibit a wide range of pharmaceutical properties including anti-atherogenic, anti-inflammatory, antitumor, and antioxidant activities [44].

#### 2.2.3. Limonoids/Coumarins

Limonoids (abundant in lemon) belonging to tetranortriterpenes were annotated in peaks 48, 49, and 54 as glycosidic conjugates and in peak 65 as the aglycone “limonin”, eluting much later at a relatively less polar eluent composition. This the first report of methyllimonexic acid in *C. limon*. Aside from their bitterness, imparting their taste to lemon, limonoids are regarded as an important constituent in food diets including *Citrus* fruit owing to their anti-obesity and anti-hyperglycemic effects [45]. Several coumarins (furano-type) were also detected, associated with peaks 56 (*m*/*z* 201.0186 [M − H]^−^), 62 (*m*/*z* 371.1488 [M − H]^−^), and 75 (*m*/*z* 297.1519 [M − H]^−^) and annotated as bergaptol, 6′,7′-dihydroxybergamottin, and geranyloxycoumarin, respectively. Lipophilic coumarins acylated with a fatty acid group and eluting in the fatty acid region were also assigned in peaks 44, 45, and 47. Except for bergapten and dihydrobergamotin octanal acetal, these coumarins are annotated for the first time in *C. limon* and might also account for the food properties or health effects observed in grapefruit.

**Figure 3:**
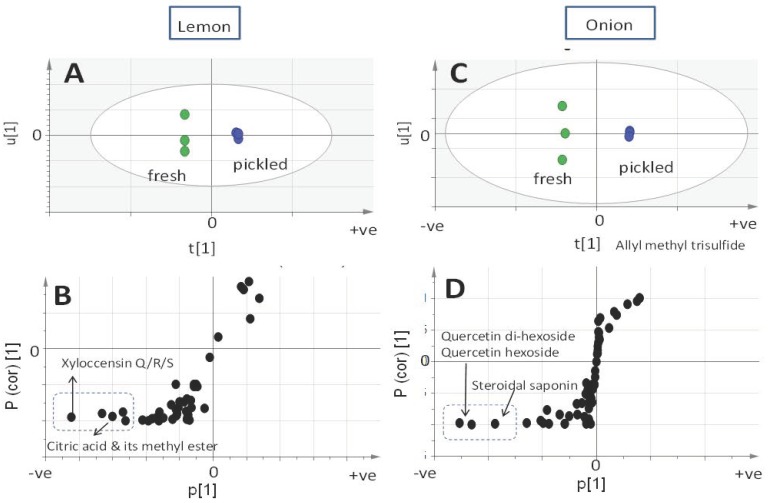
OPLS-DA score plot derived from modeling primary and secondary metabolites analyzed using UPLC-QTOFMS. Metabolites were derived fresh and pickled lemon fruit (**A**) and onion bulb (**C**), each modeled one at a time. The respective *S*-plots, (**B**) and (**D**), show the covariance p[1] against the correlation p(cor)[1] of the variables of the discriminating component of the OPLS-DA model. Cut-off values of *p* < 0.05 were used; selected variables are highlighted in the *S*-plot and identifications are discussed in the text.

The UPLC-QTOFMS dataset was further subjected to multivariate data analysis to help determine which analytical platform provides a better prediction of pickling impact on *Allium* and *Citrus* metabolism. PCA was initially constructed to reveal for the metabolites variation among fresh and pickled specimens based on the abundance of peaks. The PCA score plots (Figure S3a,b) demonstrated a clear segregation between the fresh and pickled product in both lemon and onion with a total covered variance of 86% and 94%, respectively. The OPLS-DA analysis was further employed to identify metabolites that were highly correlated with the sample type. Compared to GC-MS, the UPLC-QTOFMS-derived OPLS model was found to be more predictive of the assessment of pickling, as revealed from the prediction power of their respective models. However, it should be noted that in contrast to the GC-MS-based model, the identified variant masses in case of UPLC-QTOFMS were based on MS spectral interpretation and not confirmed with authentic standards. A clear separation was detected from the OPLS-DA score plot (Figure 3A,C) for lemon and onion. The S-loading plot of lemon (Figure 3B) revealed the presence of xyloccensin, citric acid, and its methyl ester as the most distinctive markers for fresh lemon. In contrast, onion fresh bulb was discriminated from the pickled one by the abundance of steroidal saponins in addition to quercetin glycosidic conjugates (Figure 3D). Xyloccensin exhibits an anti-hyperglycemic activity [46] and a decrease in its levels could ultimately affect lemon benefits. Moreover, the benefits of *Allium’s* polyphenols, i.e., quercetin glycoside and diglycoside, are well recognized [47] and a decrease in their levels was also observed in the case of *A. cepa* red cv. examined herein. Whether these metabolites get solubilized in the brine solution surrounding the bulb or get degraded has yet to be determined.

To assess the validity of the UPLC-MS based models, Q2 and R2 values of all calculated models were found to be larger than 0.5 and close to 1. The permutation diagnostic analysis of 20 iterations provided a reference distribution of R2/Q2 values and hence indicated the statistical significance of these parameters, with most models showing a regression line crossing zero, with negative Q2 and R2 values close to 1, which signifies the model validation. Also, the *p*-value for each OPLS-DA model was calculated using CV-ANOVA and all *p*-value were below 0.005 in the pickled models of lemon and onion (Supplementary Figures S4 and S5). Nevertheless, the models derived from the volatiles GC-MS dataset exhibited lower model validation, showing smaller R2 and Q2 values and with *p*-values higher than 0.05, suggesting that the UPLC-MS-derived models provided a better assessment of the pickling impact on both foods

### 2.3. Onion and Lemon Primary Metabolite Profiling Using GC-MS

**Figure 4:**
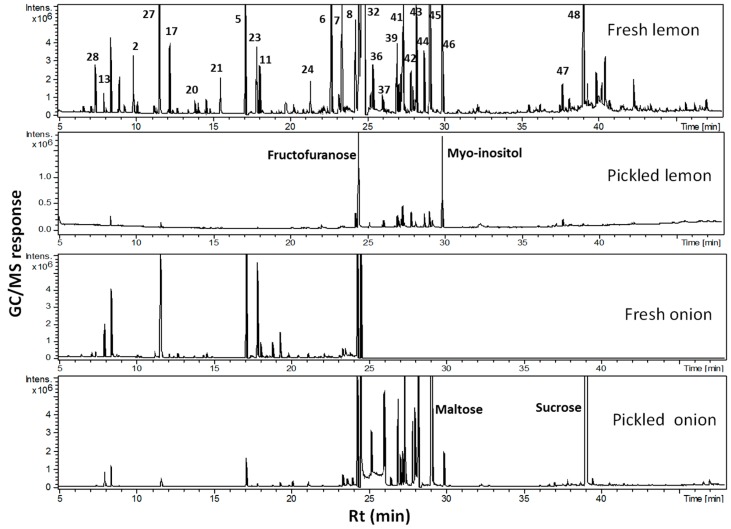
GC-MS chromatogram of silylated primary metabolites analyzed from fresh and pickled onion bulb and lemon fruit.

Although UPLC-QTOFMS revealed the variation in organic acids, viz. citric acid and its methyl derivative, as a major contributor to the segregation of pickled lemon from fresh ones, such a platform is not optimized for primary metabolite profiling [48]. To provide a more sensitive analysis of primary metabolites, viz. sugars and amino acids, compared to reversed-phase UPLC-QTOFMS, GC-MS was adopted for primary metabolite profiling in extracts prepared from fresh and pickled tissues. GC-MS analysis detected 52 primary metabolites belonging to organic acids, amino acids, fatty acids, sugars, and inorganic and nitrogenous compounds (Figure 4, Table S1). Primary metabolite analysis revealed a remarkable decrease in the total amount of organic acids, amino acids, and inorganic and nitrogenous compounds in the pickled tissue of both onion and lemon. In contrast, sugars and fatty acids were the major primary metabolites in pickled samples. Particularly in onion, fresh tissue was rich in malic acid (12.6%), proline (7%), fructofuranose (27%), and mannopyranose (11.6%). On the other hand, pickled onion showed dominancy in sugars, viz. maltose (16.3%) and sucrose (38%). In lemon, fresh tissue was also predominated by organic acids, viz. citric acid (37%), and sugar, viz. sucrose (28%), accounting for the sour taste of lemon concurrent with an abundance of sugar alcohol, viz. myo-inositol (21.9%) and fructofuranose (47%). A decrease in citric acid and its methyl ester levels in pickling can explain the less sour taste observed in pickled lemon and is in agreement with the UPLC-QTOFMS results. However, citric acid is considered a natural preservative that aids in smooth digestion and helps dissolve kidney stones [49]. Also of interest is the different sugar profile in the pickled product being more dominated by sugar alcohols, viz. myo-inositol, and monosaccharides, viz. fructose, compared to disaccharides in the fresh product, viz. sucrose. A decrease in disaccharide levels upon pickling in lemon could be attributed to the chemical hydrolysis of sucrose upon storage and especially due to the acidic nature of lemon, as an such effect was not observed in case of pickling *Allium* bulb. Sugar alcohols exhibit higher thermal stability and do not undergo any Milliard reaction compared to sugars [50].

## 3. Materials and Methods

### 3.1. Plant Material

*Allium sativum* and *A. cepa* red cv. bulbs were collected fresh from the field at Siwa Oasis, Egypt during the month of May 2016. Lemon fruit was collected at the ripe stage from trees grown in Behira governorate, Egypt during the month of May 2016.

### 3.2. Pickling Protocol

Preparing pickled lemon and onion was made by packing directly with acidified brine after blanching. Before processing, defective fruits or bulbs were discarded. Lemon fruit and pealed onion bulbs were blanched separately in water at 90 °C for 5 min and immediately packed in dark glass jars that were closed tight, containing acidified brine solution using 5% acetic acid. Brine was prepared by dissolving NaCl in water at a concentration of 15%. Both pickled products were stored in a dark place away of light at room temperature for four weeks.

### 3.3. Chemicals and Fibers

SPME fiber of stableflex coated with divinylbenzene/carboxen/polydimethylsiloxane (DVB/CAR/PDMS, 50/30 µm) was purchased from Supelco (Oakville, ON, Canada). All chemicals and standards were purchased from Sigma Aldrich (St. Louis, MO, USA). Acetonitrile and formic acid (LC–MS grade) were obtained from J.T. Baker (Avantor, Netherlands). MilliQ water was used for UPLC-PDA-ESI-TOF-MS analysis.

### 3.4. Headspace Volatiles Analysis of C. limon and A. cepa Bulbs

The headspace-solid phase microextraction (HS-SPME) volatile analysis was carried out as mentioned previously [28]. (*Z*)-3-hexneyl acetate, dissolved in water to make a final concentration of 2 µg per 1.5-mL SPME screw cap vial, was used to spike 100 mg of dried, finely ground peeled bulbs of onion and lemon fruit placed in 1.5-mL glass vials. The SPME fiber was soaked in a vial containing plant material and placed in an oven of 50 °C for 30 min. The fiber was successively withdrawn into the needle and then injected into the injection port of the gas chromatography-mass spectrometer (GC-MS). A system blank containing no plant material was run as a control. SPME fibers were desorbed at 210 °C for 1 min in the injection port of a Shimadzu Model GC-17A gas chromatograph interfaced with a Shimadzu model QP-5000 mass spectrometer (Kyoto, Japan). Volatiles were separated on a DB5-MS column (30 m length, 0.25 mm inner diameter, and 0.25 μm film) (J & W Scientific, Santa Clara, CA, USA). Injections were made in the splitless mode for 30 s. The gas chromatograph was operated under the following conditions: injector 220 °C, column oven 38 °C for 3 min, then programmed at a rate of 12 °C/min to 180 °C, maintained at 180 °C for 5 min, and finally ramped at a rate of 40 °C min^−1^ to 220 °C and maintained for 2 min. He was used as the carrier gas at 1 mL/min^−1^. The transfer line and ion-source temperatures were adjusted to be 230 and 180 °C, respectively. The HP quadrupole mass spectrometer was operated in the electron ionization mode at 70 eV. The scan range was set at *m*/*z* 40–500. Peaks were deconvoluted using AMDIS software (Gaithersburg, MD, USA www.amdis.net). Volatile components were identified by matching their retention indices (RI) relative to n-alkanes (C_6_–C_20_), as well as mass comparison to NIST and WILEY library databases and with standards.

### 3.5. GC-MS Analysis of Silylated Primary Metabolites in Fruit Pulp and Skin

Freeze-dried fruit or bulb powders (100 mg) were extracted by adding 5 mL 50% MeOH. Then, 100 μL of this 50% aqueous extract was evaporated under nitrogen until dryness was reached. Next, 150 μL of *N*-methyl-*N*-(trimethylsilyl)-trifluoroacetamide (MSTFA) was added and incubated at 60 °C for 45 min for derivatization. The primary metabolites were analyzed using GC-MS. Silylated products were purified on an Rtx-5MS (30 m length, 0.25 mm inner diameter, and 0.25 μm film) column. Injections were made in a (1:15) split mode with the following conditions: injector 280 °C, column oven 80 °C for 2 min, rate 5 °C/min to 315 °C, maintained at 315 °C for 12 min. He was used as the carrier gas at 1 mL/min^−1^. The transfer line and ion-source temperatures were set to 280 and 180 °C, respectively. Compounds were identified as described in [51].

### 3.6. Extraction Procedure and Sample Preparation for UPLC-PDA-MS Analysis

Dried lyophilized food material was grounded separately in a mortar with liquid nitrogen. A powdered aliquot (ca. 30 mg) was then homogenized with 2.5 mL 70% MeOH containing 5 mg/mL umbelliferone (internal standard) with sonication for 30 min. Extracts were vortexed, centrifuged at 3000× *g* for 30 min to remove debris, and stored. Chromatographic separation was performed on an Acquity UPLC system (Waters Corp., Milford, MA, USA) equipped with an HSS T3 column (100 × 1.0 mm, particle size 1.8 mm; Waters Corp.) applying the following binary gradient at a flow rate of 150 mL/min: 0 to 1 min, isocratic 95% A (water:formic acid, 99.9:0.1, *v*/*v*), 5% B (acetonitrile:formic acid, 99.9:0.1, *v*/*v*); 1 to 16 min, linear from 5 to 95% B; 16 to 18 min, isocratic 95% B; 18 to 20 min, isocratic 5% B.

### 3.7. GC-MS and UPLC-QTOFMS Data Processing for Multivariate Analysis

MS peak abundance of volatiles and secondary metabolites were extracted using MET-IDEA software with default parameter settings for GC-MS and LC-MS files [52]. The aligned peak abundance data table was normalized to a spiked internal standard and further exported to principal component analysis (PCA) and partial least squares-discriminant analysis (OPLS-DA) using SIMCA-P version 13.0 software package (Umetrics, Umeå, Sweden). All variables were mean-centered and scaled to Pareto variance.

## 4. Conclusions

Pickling using brine, an old traditional process used to preserve food products, was investigated for the first time using an MS-based metabolomics approach in two food materials—lemon and onion. Metabolomics was effectively implemented to highlight the similarities and differences between fresh and pickled products. Chemical analysis revealed a decline in lemon sensory volatiles, i.e., α,β-citral, as well as the degradation and rearrangement of sulphur compounds in onion to generate 2-hexyl-5-methyl-3(2*H*)-furanone and 3,5-diethyl-1,2,4-trithiolane, which are sulphur rearrangement products. A marked decrease in citric acid levels in lemon is likely to account for the less sour taste of its fruit. Nevertheless, it should be noted that the chemical changes observed in this study need to be further complemented with a sensory analysis to prove whether they are ultimately reflected in changes in aroma or taste. The impact of pickling on the non-volatile polar bioactive metabolites was revealed via UPLC-QTOFMS analysis and suggested for a lower health value of pickled product relative to the fresh specimens. The effect of blanching as part of the pickling was also not separately assessed, as samples were only analyzed after the pickling step.

## Figures and Tables

**Table 1:** Relative percentiles of volatile components to total peak areas detected in fresh and pickled lemon fruit using SPME/GC-MS measurements (*n* = 3).

Peak	rt (min)	KI	ID	Average ± Std. dev. Fresh	Average ± Std. dev. Pickled
1	7.03	903	α-Thujene	0.15 ± 0.11	0.04 ± 0.03
2	7.18	912	α-Pinene ^a^	0.37 ± 0.48	0.35 ± 0.27
3	7.48	929	Fenchene	0.04 ± 0.04	0.02 ± 0.02
4	8.03	961	β-Pinene ^a^	33.24 ± 5.89	23.44 ± 1.26
5	8.16	967	β-Myrcene ^a^	0.15 ± 0.20	0.36 ± 0.28
6	8.64	995	1,4-Cineole ^a^	0.02 ± 0.02	0.07 ± 0.06
7	8.66	996	2-Carene	2.12 ± 2.69	0.17 ± 0.13
8	8.80	1005	Melilotal	0.05 ± 0.07	0.39 ± 0.31
9	8.94	1014	Limonene ^a^	32.10 ± 10.45	43.49 ± 5.16
10	9.38	1042	γ-Terpinene	12.39 ± 8.90	20.97 ± 3.21
11	9.76	1066	Terpinolene	0.10 ± 0.13	0.52 ± 0.41
12	9.86	1073	p-Cymenene	0.02 ± 0.03	0.19 ± 0.15
13	10.43	1110	α-Linalool ^a^	0.01 ± 0.02	0.37 ± 0.29
14	11.06	1154	Bicyclo[3.2.1]oct-3-en-2-one, 4-methyl-	0.01 ± 0.02	0.36 ± 0.29
15	11.10	1158	α-Phellandren-8-ol	0.02 ± 0.03	0.03 ± 0.02
16	11.22	1166	1-Terpinen-4-ol	0.99 ± 0.81	0.89 ± 0.71
17	11.33	1173	p-Cymen-8-ol	0.02 ± 0.03	0.05 ± 0.04
18	11.43	1180	α-Terpineol	0.39 ± 0.49	3.33 ± 2.66
19	11.56	1189	Unknown	0.15 ± 0.18	2.50 ± 2.00
20	11.97	1220	β-Citral	3.46 ± 4.47	0.00 ± 0.00
21	12.36	1249	α-Citral ^a^	10.86 ± 11.79	0.00 ± 0.00
22	13.16	1311	δ-EIemene	0.02 ± 0.03	0.08 ± 0.07
23	13.37	1328	6-Dimethyl-2,6-octadien-8-yl acetate	0.70 ± 0.90	0.24 ± 0.19
24	14.28	1402	(*Z*)-β-Farnesene	0.33 ± 0.46	0.23 ± 0.18
25	14.34	1407	(*E*)-α-Bergamotene	2.29 ± 2.56	0.25 ± 0.20
26	14.70	1437	Unknown sesquiterpene	0.00 ± 0.00	0.01 ± 0.01
27	15.04	1464	Unknown sesquiterpene	0.00 ± 0.00	0.05 ± 0.04
28	15.08	1468	(*Z,E*)-α-Farnesene	0.02 ± 0.03	0.80 ± 0.63
29	15.12	1478	β-Bisabolene	0.01 ± 0.01	0.69 ± 0.54
30	15.80	1523	epi-α-Eudesmol	-	0.01 ± 0.01
31	16.06	1541	Unknown sesquiterpene	-	0.08 ± 0.07
			Total monoterpene hydrocarbons %	80.66	89.55
			Total oxygenated monoterpenes %	15.83	5.49
			Total sesquiterpene hydrocarbons %	3.37	2.44
			Total oxygenated sesquiterpenes %	0.7	0.25

^a^ denotes volatiles confirmed using the authentic standard in addition to RI and MS spectral matching.

**Table 2:** Relative percentiles of volatile components to total peak areas detected in fresh and pickled onion bulb using SPME/GC-MS measurements (*n* = 3).

Peak	rt (min)	KI	Name	Average ± Std. dev. Fresh	Average ± Std. dev. Pickled
1	7.44	924	1-propenyl methyl disulphide	2.91 ± 2.19	-
2	7.99	958	Dimethyl trisulfide ^a^	3.24 ± 3.00	2.31 ± 3.18
3	9.79	1068	2-Acetylpyrrole	0.26 ± 0.15	0.00 ± 0.00
4	10.12	1089	Unknown sulphur	3.45 ± 0.36	6.71 ± 1.27
5	10.31	1101	Isopropyl α-mercaptopropionate	10.42 ± 1.94	8.35 ± 0.70
6	10.89	1143	Diethanol disulphide	12.20 ± 5.82	10.90 ± 2.89
7	10.99	1149	Methyl pentyl disulfide	0.24 ± 0.10	-
8	11.11	1157	Allyl methyl trisulfide	11.48 ± 5.08	25.25 ± 7.56
9	11.83	1209	β-Hydroxyethyl phenyl ether	0.38 ± 0.20	-
10	11.88	1212	Dimethyl tetrasulfide	3.35 ± 3.67	-
11	12.16	1234	3-Isopropylbenzaldehyde	0.26 ± 0.05	-
12	12.32	1246	4,7-Dimethylundecane	0.20 ± 0.10	-
13	12.92	1292	(Allylsulfanyl)acetonitrile	2.72 ± 3.67	-
14	13.23	1316	Dipropyl trisulfide	4.44 ± 1.26	1.28 ± 2.21
15	13.37	1328	3,5-Diethyl-1,2,4-trithiolane	18.95 ± 7.15	32.08 ± 4.53
16	13.42	1332	Diallyl trisulfide isomer	0.75 ± 0.42	-
17	14.43	1415	2-Hexyl-5-methyl-3(2*H*)-furanone	0.42 ± 0.43	3.44 ± 1.06
18	15.41	1495	Unknown hydrocarbon	0.34 ± 0.29	-
19	16.76	1589	4,6-Diethyl-1,2,3,5-tetrathiolane	10.03 ± 9.17	9.69 ± 1.13
20	17.00	1605	2,4-Dimethyl-5,6-dithia-2,7-Nonadienal	13.96 ± 12.73	-
			Total sulphur compounds %	98.14	96.57

^a^ denotes volatiles confirmed using the authentic standard in addition to RI and MS spectral matching.

**Table 3:** Peak annotations of metabolites in fresh *C. limon* fruit methanol extract using UPLC-PDA-QTOFMS in negative- and positive-ionization modes.

Peak	rt (min)	M + H/M − H	Error (ppm)	Formula	MSMS	Name	Class
1	0.51	339.1088	2.909	C_12_H_21_O_11_^−^	-	Methyl-*O*-hexosyl pentoside	Sugar
2	0.66	381.078958	−2.67	C_17_H_17_O_10_^+^	-	Hydroxybergaptol hexoside	Coumarin
3	0.67	191.01953	0.9	C_6_H_7_O_7_^−^	111	(Iso)citric acid	Organic acid
4	0.96	205.0351	0.82	C_7_H_9_O_7_^−^	173, 111	Methylated (iso)citric acid	Organic acid
5	8.74	375.09094	−1.25	C_15_H_19_O_11_^+^	357, 339, 231, 137	Unknown	
6	8.95	357.11874	0.73	C_16_H_21_O_9_^−^	195	*O*-Feruloyl-hexoside	Phenolic acid
7	9.03	595.16473	−1.02	C_27_H_31_O_15_^+^	449, 287	Luteolin *O*-rhamnosyl-hexoside	Flavone-*O*-glycoside
8	9.31	625.17462	−1.69	C_28_H_33_O_16_^+^	607, 589, 487	Diosmetin-*C*,*C*-di-hexoside	Flavone-*C*-glycoside
9	9.72	565.15454	−0.64	C_26_H_29_O_14_^+^	433, 415, 313	Apigenin-*C*-hexosyl-*O*-pentoside	Flavone-*C*-glycoside
10	9.85	433.11185	−1.07	C_21_H_21_O_10_^+^	415, 367, 313	Apigenin-*C*-hexoside	Flavone-*C*-glycoside
11	10.1	463.12177	−1.72	C_22_H_23_O_11_^+^	445, 301	Diosmetin-*C*-hexoside	Flavone-*C*-glycoside
12	10.23	625.17487	−1.44	C_28_H_33_O_16_^+^	-	Diosmetin-*C*,*C*-di-hexoside isomer	Flavone-*C*-glycoside
13	10.23	579.16962	−1.21	C_27_H_31_O_14_^+^	433, 271	Apigenin-*O*-rhamnosyl-hexoside	Flavone-*O*-glycoside
14	10.33	609.1799	−1.41	C_28_H_33_O_15_^+^	463. 301	Diosmetin-*O*-rhamnosyl-hexoside	Flavone-*O*-glycoside
15	10.43	611.19482	−2.23	C_28_H_35_O_15_^+^	465, 449, 303	Hesperetin-*O*-rhanmosyl hexoside	Flavanone-*O*-glycoside
16	10.63	395.09641	−0.86	C_18_H_19_O_10_^+^	377, 291, 147	Hydroxybergaptol-*O*-methyl ether hexoside	Coumarin
17	10.71	509.1282	−0.77	C_23_H_25_O_13_^+^	347	Limocitrin-*O*-hexoside	Flavonol-*O*-glycoside
18	10.8	797.21155	−1.93	C_35_H_41_O_21_^+^	433, 347	Limocitrin-*O*-glycosyl malonate	Flavonol
19	10.82	767.20105	−1.87	C_34_H_39_O_20_^+^	317	Gossypetin rhamnosyl dideoxyhexosyl hexoside	Flavonol-*O*-glycoside
20	10.84	717.22156	−2.1	C_31_H_41_O_19_^+^	391, 255	Xyloccensin Q/R/S	Limonoid
21	10.97	687.21094	−2.15	C_30_H_39_O_18_^+^	669, 303	Unknown hesperitin glycoside	Flavanone-*O*-glycoside
22	11.06	653.1687	−2.53	C_29_H_33_O_17_^+^	347	Unknown	Flavonol-*O*-glycoside
23	11.13	479.11685	−1.55	C_22_H_23_O_12_^+^	461, 287	Luteolin dimethyl ether-*O*-hexoside	Flavone-*O*-glycoside
24	11.20	371.1485	−2.087	C_17_H_23_O_9_^−^	-	Citrusin E	Phenolic acid
25	11.26	305.10092	−1.04	C_16_H_17_O_6_^+^	203	Unknown	
26	12.00	345.06082	0.33	C_17_H_13_O_8_^−^	330	Limocitrin	Flavonol
27	12.39	247.05951	−0.59	C_13_H_11_O_5_^+^	232	Unknown	
28	12.75	515.19141	0.24	C_27_H_31_O_10_^−^	469	Methyllimonexic acid	Limonoid
29	12.78	471.19974	−1.6	C_26_H_31_O_8_^+^	425,409, 367	Limonine	Limonoid
30	12.97	287.0907	−0.7	C_16_H_15_O_5_^+^	203	Trichoclin	Coumarin
31	13.00	327.14435	0.52	C_16_H_23_O_7_^−^	173, 111	Unknown	
32	13.12	293.17548	0.74	C_17_H_25_O_4_^−^	236, 221	Unknown terpene	Terpene
33	13.13	515.22675	−0.81	C_28_H_35_O_9_^+^	455, 411, 393, 369	Nomilinic acid	Limonoid
34	13.54	455.20485	−1.6	C_26_H_31_O_7_^+^	437, 409, 393	Deoxylimonin/obacunone	Limonoid
35	13.55	269.08	0.95	C_16_H_13_O_4_^−^	214, 201	Unknown terpene	Terpene
36	13.59	309.21	−0.05	C_18_H_29_O_4_^−^	291, 251	Unknown	
37	13.67	299.0924	1.04	C_17_H_15_O_5_^−^	284	Trihydroxy-dimethylflavanone	Flavanone
38	14.21	233.04378	−0.67	C_12_H_9_O_5_^+^	218, 205, 173	Hydroxybergapten	Coumarin
39	14.27	301.10632	−0.73	C_17_H_17_O_5_^+^	233	Hydroxy-dimethoxyflavanone	Flavanone
40	14.27	339.0621	−3.09	C_22_H_11_O_4_^+^	-	Unknown	
41	15.00	313.14386	0.42	C_19_H_21_O_4_^−^	176	Geranyloxy hydroxy coumarin.	Fatty acyl coumarin
42	15.45	481.2717	−5.06	C_29_H_37_O_6_^−^	-	Dihydrobergamotin octanal acetal	Coumarin
43	15.65	377.11435	−2.87	C_26_H_17_O_3_^+^	241	Unknown	
44	16.07	369.16898	−0.67	C_22_H_25_O_5_^+^	233	Hydroxybergaptol-*O*-dimethyl-octadienyl-Me ether	Fatty acyl coumarin
45	16.17	339.15781	−1.28	C_21_H_23_O_4_^+^	203	Bergaptin	Fatty acyl coumarin
46	16.19	337.14368	0.24	C_21_H_21_O_4_^−^	267, 254	Geranyloxypsoralen (bergaptin)	Coumarin
47	16.22	329.17377	−0.97	C_20_H_25_O_4_^+^	193, 184	Geranyloxy-methoxycoumarin	Fatty acyl coumarin
48	16.30	271.2276	0.71	C_16_H_31_O_3_^−^	225	Hydroxypalmitic acid	Fatty acid
49	16.52	339.19971	−1.63	C_15_H_31_O_8_^−^	183	Unknown	Fatty acid

## Supplementary Materials

The followings are available online, Table S1: Relative percentiles of silylated primary metabolites detected in fresh and pickled onion bulb and lemon fruit using GC-MS measurements; Figure S1: SPME/GC-MS-based PCA score plot derived from modeling the pickling effect on lemon fruit to assess the effect of pickling on metabolite composition; Figure S2. UPLC-QTOFMS chromatogram of metabolites analyzed from fresh and pickled lemon fruit (A) and onion bulb (B) in negative- and positive-ionization modes, showing qualitative differences upon pickling in each examined food; Figure S3: UPLC-QTOFMS-based PCA score plot derived from modeling the pickling effect on lemon fruit (A) and onion bulb red cv. (B) separately to assess the effect of pickling on metabolite composition.
