# Supplementary material for: Influence of Pickling Process on Allium cepa and Citrus limon Metabolome as Determined via Mass Spectrometry-Based Metabolomics
Source: Molecules. 2019 Mar 7;24(5):928. doi: 10.3390/molecules24050928 (PMC6429351; doi:10.3390/molecules24050928)

Suppl. Table S1 Relative percentile level of silylated primary metabolites detected in fresh and pickled onion bulb and lemon fruit using GC–MS measurements (n = 3).

	**RT**	**KI**	**NAME**	**Class**	**Onion fresh**	**Onion pickled**	**Lemon fresh**	**Lemon pickled**
1	6.39	1055	Lactic acid (2TMS)	acid	0.54	0.08	0.06	0.25
2	9.87	1203	Malonic acid (2TMS)	acid	0.05	0.01	0.60	3.88
3	13.10	1329	Glyceric acid (3TMS)	acid	0.03	0.01	0.01	0.00
4	14.08	1367	Unknown acid	acid	0.08	0.10	0.15	4.32
5	17.08	1486	Malic acid (3TMS)	acid	12.55	1.38	4.55	0.00
6	22.70	1729	Citric acid (4TMS)	acid	0.03	0.00	1.46	0.00
7	23.38	1761	Citric acid (4TMS)	Acid	0.06	0.00	1.96	0.01
8	24.56	1815	Citric acid (4TMS)	acid	3.38	0.22	20.04	0.10
9	24.76	1819	Citric acid (4TMS)	acid	0.19	0.32	13.46	0.01
10	12.73	1315	Succinic acid (2TMS)	acid	0.15	0.07	0.03	0.00
11	18.06	1526	γ-Aminobutyric acid (3TMS)	acid	0.39	0.00	0.18	0.00
12	18.85	1560	Threonic acid (3TMS)	acid	0.54	0.03	0.02	0.00
				**Total acids**	**17.97**	**2.23**	**42.52**	**8.57**
13	7.88	1122	Ethylene glycol (2TMS)	Alcohol	1.00	0.23	0.31	0.01
				**Total alcohols**	**1.00**	**0.23**	**0.31**	**0.01**
14	7.42	1103	Alanine (2TMS)	amino acid	0.74	0.07	0.59	0.00
15	10.13	1213	Valine (2TMS)	amino acid	0.33	0.02	0.13	0.01
16	11.21	1255	Serine (2TMS)	amino acid	0.28	0.02	0.08	0.00
17	12.14	1291	Threonine (2TMS)	amino acid	0.17	0.01	0.02	0.08
18	12.24	1295	Proline (2TMS)	amino acid	0.01	0.00	0.68	0.00
19	12.42	1302	Glycine (2TMS)	amino acid	0.13	0.01	0.06	0.00
20	13.41	1341	Cadaverine (4TMS)	amino acid	0.12	0.00	0.02	0.00
21	15.52	1423	Aspartic acid (2TMS)	amino acid	0.05	0.00	0.28	0.00
22	17.84	1517	Proline, 5-oxo-(2TMS)	amino acid	7.03	0.16	0.60	0.00
23	17.89	1519	Aspartic acid, N-(3TMS)	amino acid	0.10	0.00	0.85	0.00

24	21.30	1666	Asparagine (3TMS)	amino acid	0.01	0.00	0.13	0.01
				**Total amino acids**	**8.96**	**0.30**	**3.43**	**0.12**
25	32.14	2197	Linoleic acid (TMS)	fatty acid	0.00	0.02	0.03	0.67
26	32.25	2202	Oleic acid (TMS)	fatty acid	0.00	0.00	0.00	0.00
				**Total fatty acids**	**0.00**	**0.03**	**0.03**	**0.68**
27	11.57	1269	Phosphate (3TMS)	inorganic	12.99	0.51	1.94	0.00
				**Total inorganic**	**12.99**	**0.51**	**1.94**	**0.00**
28	7.17	1091	*N*-Ethanolamine (2TMS)	nitrogenous compound	0.54	0.02	0.06	0.01
				**Total nitrogenous**	**0.54**	**0.02**	**0.06**	**0.01**
29	19.29	1579	Xylulose (4TMS)	sugar	0.76	0.07	0.00	0.00
30	21.11	1657	1,5-Anhydro-D-sorbitol (4TMS)	sugar	0.30	0.14	0.01	0.12
31	23.35	1761	Tagatofuranose (5TMS)	sugar	1.30	0.72	0.34	0.61
32	24.22	1798	Fructofuranose (5TMS)	sugar	15.62	6.28	1.33	47.42
33	24.46	1811	Sorbose (5TMS)	sugar	8.48	4.27	2.18	0.00
34	24.54	1809	Fructofuranose (5TMS)	sugar	11.86	3.95	2.68	0.00
35	25.10	1841	Talofuranose (5TMS) (isomer 2)	sugar	1.35	0.59	0.11	0.47
36	25.17	1845	Psicopyranose (5TMS) (isomer 2)	sugar	2.71	0.91	0.07	0.48
37	26.01	1885	Psicose (5TMS)	sugar	4.45	2.48	0.11	0.26
38	26.07	1888	Mannopyranose (5TMS)	sugar	11.69	1.59	0.24	2.28
39	26.90	1931	Gluconic acid (4TMS) lactone	sugar	0.00	4.29	0.38	0.00
40	27.29	1950	Myo-Inositol (6TMS)	sugar	0.00	0.11	0.50	2.37
41	27.35	1953	Fructose (5TMS)	sugar	0.00	0.04	0.15	0.32
42	27.83	1977	Ribitol (5TMS)	sugar	0.00	2.94	0.49	4.19
43	28.22	1997	Talofuranose (5TMS) (isomer 2)	sugar	0.00	8.84	1.65	0.98
44	28.70	2021	Inositol (6TMS)	sugar	0.00	0.00	0.74	3.38
45	29.05	2040	Maltose, octakis(trimethylsilyl) ether, methyloxime	sugar	0.00	16.37	4.58	2.88
46	29.86	2081	Myo-Inositol, hexakis-O-(trimethylsilyl)-	sugar	0.00	1.42	3.43	21.88
47	37.66	2478	α-Mannobiose, octakis(trimethylsilyl) ether	sugar	0.00	0.01	0.17	1.13
48	38.90	2539	Sucrose, octakis(trimethylsilyl) ether	sugar	0.00	23.56	3.32	1.48
49	39.20	2558	Lactose, octakis(trimethylsilyl)-	sugar	0.00	0.55	0.32	0.35
50	49.71	3090	Sucrose, octakis(trimethylsilyl) ether	sugar	0.00	15.28	28.92	0.00

51	50.26	3118	Unknown disaccharide	sugar	0.00	1.25	0.00	0.00
52	53.85	3301	Unknown disaccharide	sugar	0.00	1.02	0.00	0.00
				**Total sugars**	**58.52**	**96.68**	**51.71**	**90.62**

Suppl. Fig. S1 SPME GC/MS based PCA score plot derived from modelling pickling effect on *lemon fruit* to assess the effect of pickling on metabolites composition (n = 3).

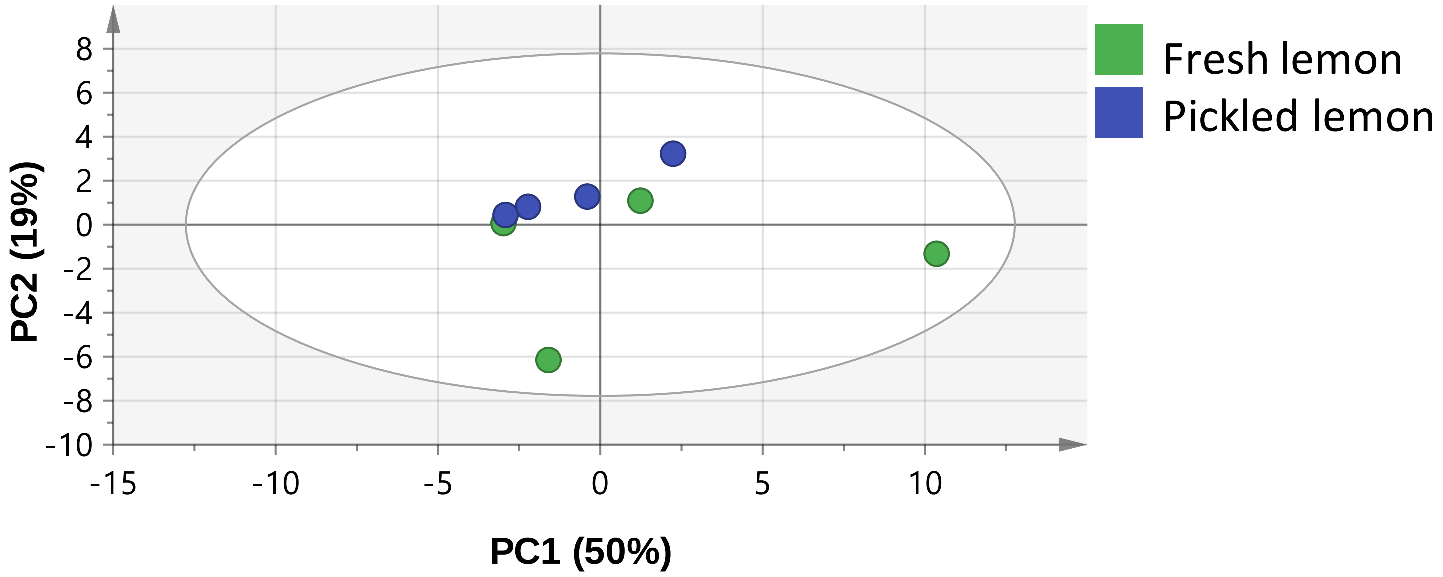

Suppl. Fig. S2

UPLC-MS chromatogram of metabolites analysed from fresh/pickled lemon fruit (**A**) and onion bulb (**B**) acquired in negative and positive ionizarion modes showing qualitative differences upon pickling in each examined food.

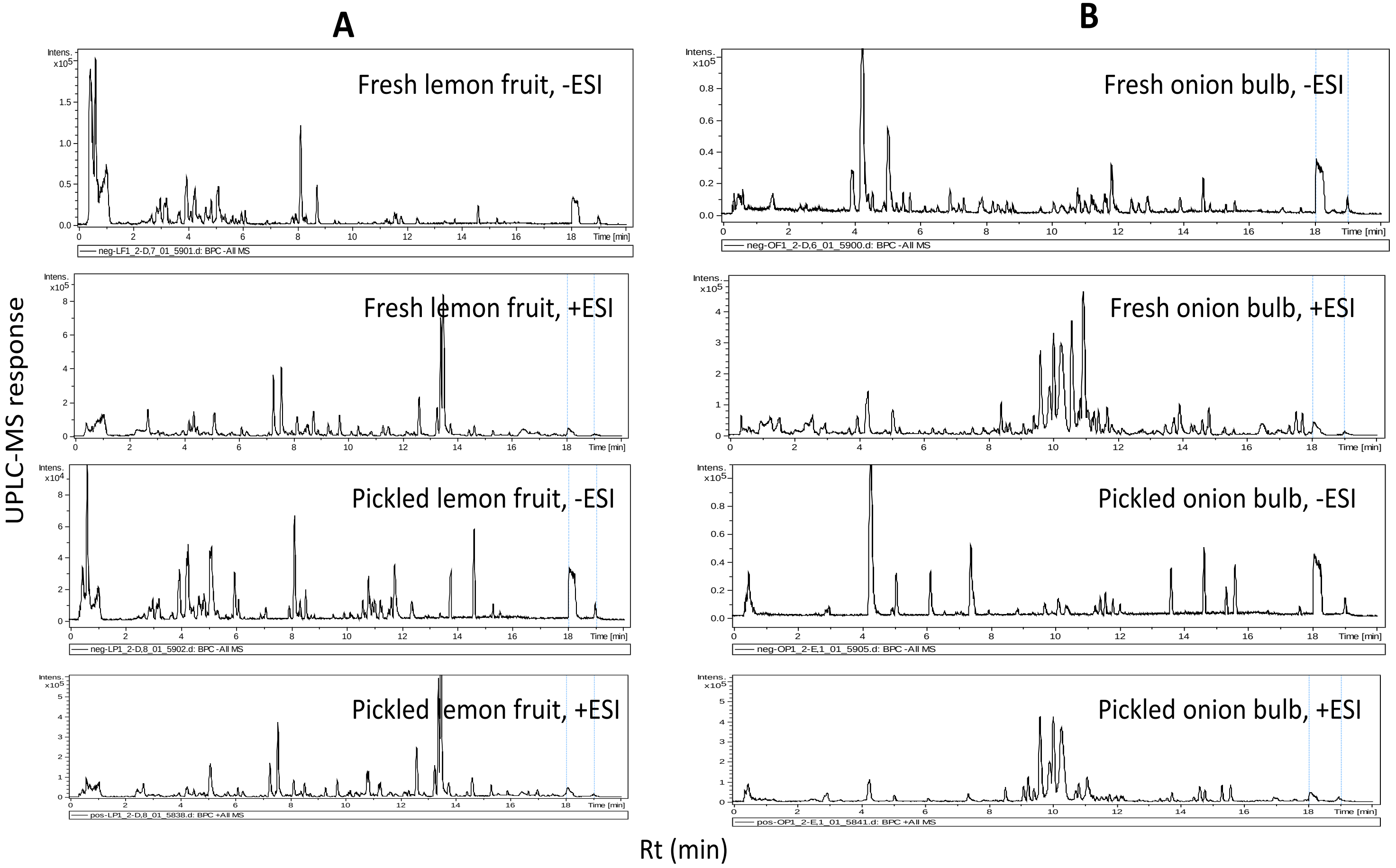

Suppl. Fig. S3 UPLC/MS based PCA score plot derived from modelling pickling effect on *lemon fruit* (A) and onion bulb red cv. (B) one at a time separately to assess the effect of pickling on metabolites composition (n = 3).

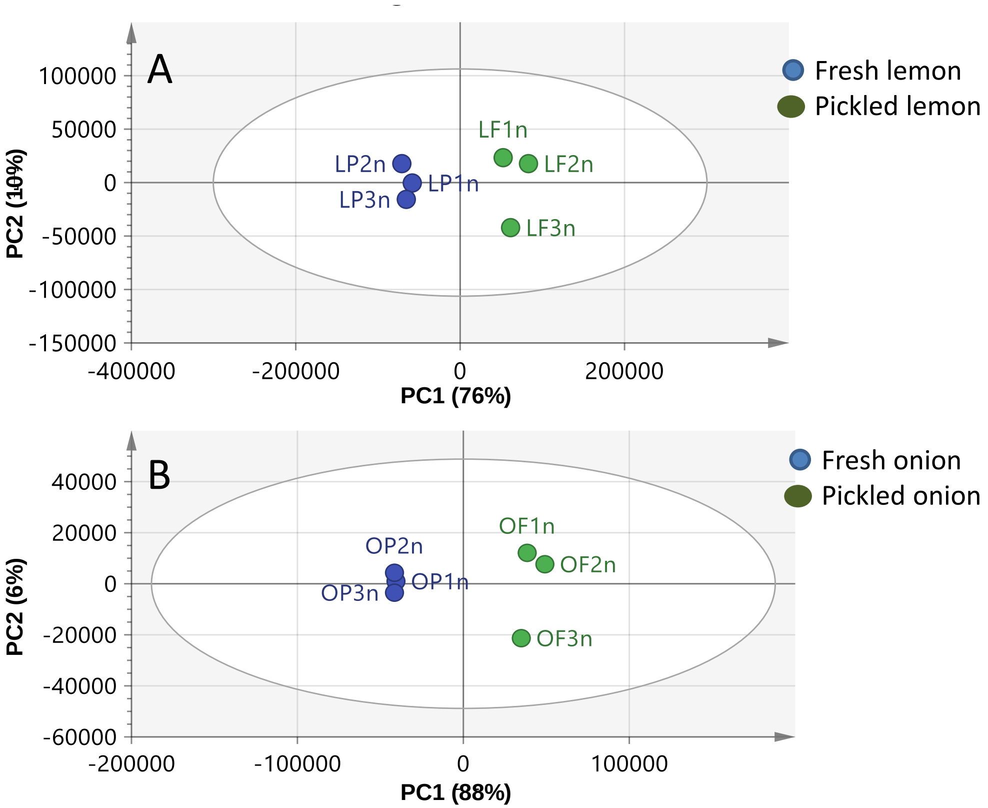

Supplement: Supplementary file 1 [file molecules-24-00928-s001.pdf]
